# Supplementary material for: Frequent PIK3CA mutations in eutopic endometrium of patients with ovarian clear cell carcinoma
Source: Mod Pathol. 2021 Jun 25;34(11):2071–9. doi: 10.1038/s41379-021-00861-3 (PMC8514336; doi:10.1038/s41379-021-00861-3)
Supplement: Supplementary file 1 — Figure S1-S4, Table S1-S2 [file 41379_2021_861_MOESM1_ESM.pdf]

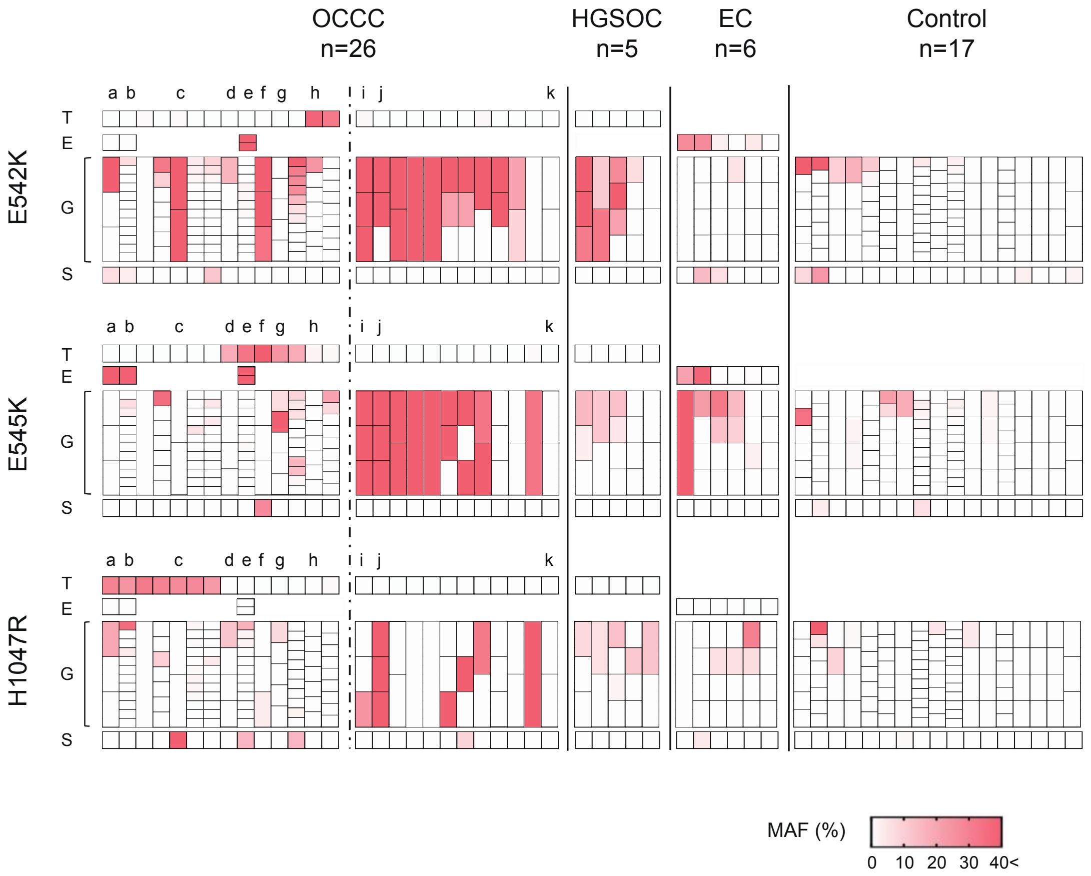

Supplementary Figure S1. Heatmap of the MAF of *PIK3CA* in OCCC, endometriosis, and endometrium in all cases. The samples are arranged as in Fig. 2A, and all MAF values are shown in the heat map. For the OCCC cases, the cases on the left side of the dotted line have *PIK3CA* mutation in the tumor, while the cases on the right side are cases without *PIK3CA* mutation. a–k show cases in which tumors were macro- or microdissected and multi-sampled (details are shown in Fig. 3). MAF: mutant allele frequency, OCCC: ovarian clear cell carcinoma, HGSOC: high-grade serous ovarian carcinoma, EC: endometriotic cyst, T: tumor, E: endometriosis, G: endometrial gland, S: endometrial stroma.

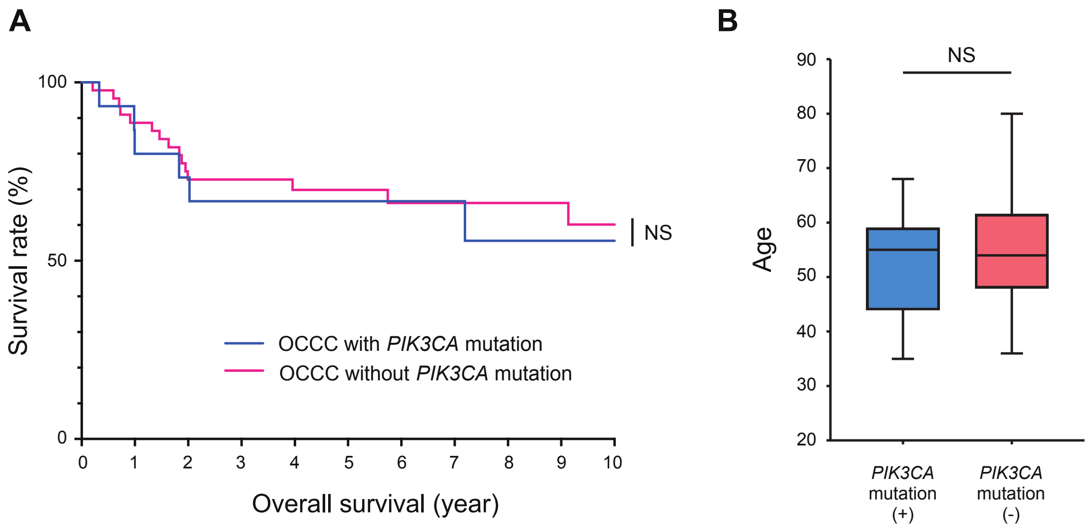

Supplementary Figure S2. Survival and age in OCCC cases according to *PIK3CA* mutation. A, Overall survival of 60 OCCC cases. Patients were divided into two groups according to the presence of *PIK3CA* mutation with MAF ≥15%. Overall survival is shown by Kaplan–Meier curve. The blue line indicates the group with MAF ≥15% of *PIK3CA* mutation, and the red line indicates the other group. B, Age. Patients were divided into two groups according to the presence of *PIK3CA* mutation with MAF ≥15%. Age of each group is shown in a box and whisker plot. NS: not significant. OCCC: ovarian clear cell carcinoma.

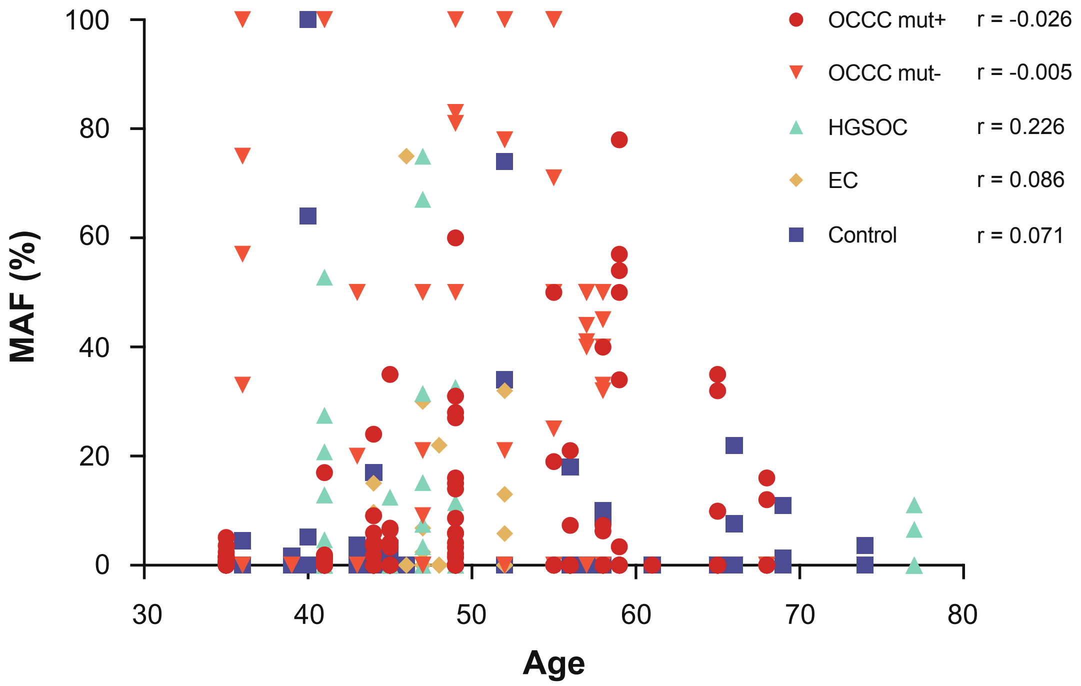

Supplementary Figure S3. Correlation between eutopic endometrial glands and age. Spearman's rank correlation coefficient was calculated for age and MAF. There was no correlation between age and MAF in the group with *PIK3CA* mutation with MAF ≥15% in the tumor of OCCC (OCCC mut+), the group without mutation (OCCC mut-), HGSOC, EC, and controls. OCCC: ovarian clear cell carcinoma, HGSOC: high-grade serous ovarian carcinoma, EC: endometriotic cyst, MAF: mutant allele frequency.

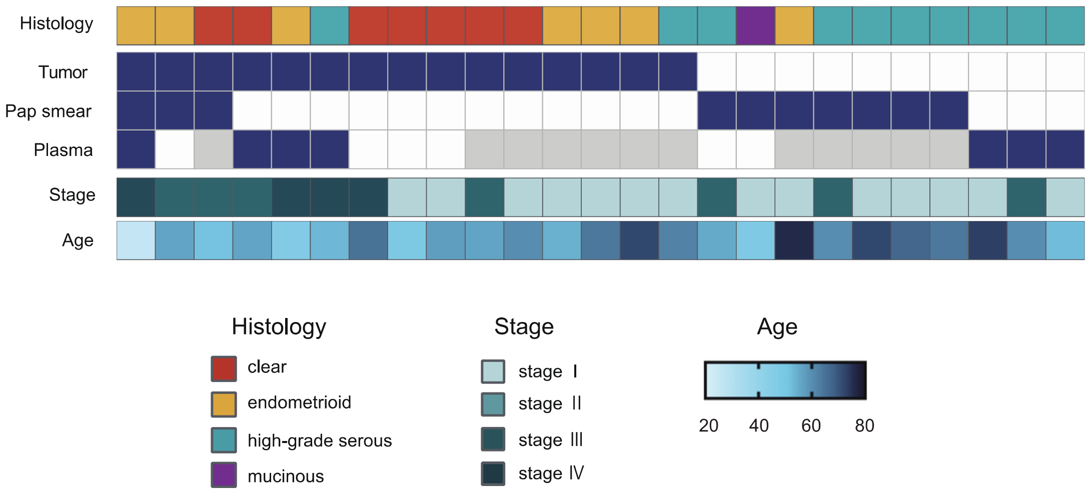

Supplementary Figure S4. Twenty-five ovarian cancer cases with *PIK3CA* mutations in tumor, Pap smear, and plasma from the study by Wang et al. Tumor, Pap smear, and plasma with *PIK3CA* mutations are shown in dark blue. Cases that did not have samples are shown in gray.

Supplementary Table S1 Toluidine blue staining

Deparaffinization with xylene
↓
Rinsing with distilled water
↓
Soaking in 0.05% toluidine blue solution for 25 seconds
↓
Remove staining solution and dehydration with anhydrous ethanol
↓
Drying in the air

Supplementaly Table S2 Thermal cycles for droplet digital PCR

Step	Tempature (°C)	Time (sec)	
1	95	600	
2	94	30 *	
3	55	60 *	*repated for 39 cycles
4	98	600	
5	4	∞	
